# Editorial: Aspiration management and rehabilitation

**DOI:** 10.3389/fresc.2025.1558680

**Published:** 2025-02-11

**Authors:** Phyllis M. Palmer, Paula Leslie

**Affiliations:** ^1^ Department of Speech & Hearing Sciences, University of New Mexico, Albuquerque, NM, United States; ^2^ Medical Physics and Clinical Engineering, Newcastle upon Tyne Hospitals NHS Foundation Trust, Newcastle, United Kingdom; ^3^ Center for Bioethics and Health Law, University of Pittsburgh, Pittsburgh, PA, United States

**Keywords:** aspiration, evidence-based clinical practice, dysphagia, risk factors, implementation science, swallowing disorders, critical reflection, myth busting

## Abstract

Clinical management of prandial aspiration remains heavily influenced by long-standing practices and may not align with current evidence. This editorial provides a broad overview of the articles in this edition of *Frontiers in Rehabilitation Sciences* and addresses three common misconceptions in dysphagia management: (a) that prandial aspiration always requires immediate restrictive intervention, (b) that coughing during meals indicates physiologic dysfunction, and (c) that thickened liquids universally reduce aspiration risk without consequence. We examine how these myths conflict with current evidence and highlight supportive perspectives from various disciplines. Rather than introducing new techniques, we encourage critical examination of current practices and provide guidance for implementing evidence-supported interventions. The goal is to move toward individualized care that considers multiple risk factors beyond the mere presence of aspiration, ultimately improving patient outcomes while maintaining quality of life.

**Editorial on the Research Topic**
Aspiration management and rehabilitation

## Introduction

In teaching future dysphagia clinicians, we observed a disconnect between the evidence taught in the classroom and real-world practice. Intervention decisions often focus on the presence or absence of aspiration, rather than critical variables such as oral care and immune function that impact clinical outcomes (Cimoli et al., Lisiecka et al., Palmer and Padilla, Ashford). Given that prandial aspiration's consequences can range from insignificance to death, implementing evidence-supported intervention is essential.

The implementation gap between research and clinical practice can span 20 or more years (1, 2). Langmore and colleagues (3) landmark study on aspiration risk factors is now well beyond that expected window, yet wide-spread adoption of evidence-based practice remains inconsistent. Clinicians face multiple barriers to employing evidence-based approaches including (a) limited time for continuing education, (b) the exponential increase in annual publications, (c) insufficient financial support for advanced training, and (d) minimal administrative support (4–6).

Beyond resource constraints lies the fundamental challenge of translating evidence into actionable clinical protocols. Without clear guidance clinicians may default to familiar practices passed down through workplace culture. Outdated approaches can become entrenched and continue to influence clinical practice patterns, particularly when resource constraints limit opportunities for professional development and critical self-reflection. The gap between knowing the evidence and applying it effectively has led to persistent underuse of evidence-based practices.

Intervention in the case of aspiration requires a multidisciplinary approach (Lisiecka et al., Okon et al.). This research topic brings together authors from diverse clinical fields, including speech language pathologists, nurses, dentists, physicians (critical care, gerontology, pulmonary disease), to break disciplinary silos. Through “myth busting” and promoting evidence-based practices, we address persistent clinical misconceptions while acknowledging real-world constraints. Our goal is to help clinicians critically examine embedded myths and transition to evidence-supported practice patterns. Rather than introducing new techniques, we encourage critical reflection on current practice and practical implementation of defensible approaches.

## Long-standing approaches to aspiration management

### Myth 1: Prandial aspiration is always dangerous

A pervasive myth is the assumption that any observed aspiration during mealtimes leads to an adverse medical outcome and requires immediate, often restrictive, interventions such as nil per os (aka, nil by mouth, NPO) status. Such thinking fails to account for the complex interplay of factors that influence aspiration-related health outcomes. Lisiecka et al. demonstrate that aspiration pneumonia, a potential risk from aspiration, is not a single-factor condition. While aspiration during mealtimes certainly warrants clinical attention, the presence of aspiration alone does not automatically predict adverse events such as pneumonia or respiratory compromise (Cimoli et al., Lisiecka et al., Palmer and Padilla, Ashford). The mapping review by Lisiecka et al., combined with frameworks proposed by Ashford and Palmer and Padilla identify crucial factors such as overall health status, immune function, oral health and hygiene, types of foods and liquids being aspirated that determine potential complications from aspiration. Dallal-York and Troche add that the ability to protect one's airway through a productive cough is important in assessing risk. This nuanced understanding supports the idea that blanket approaches, such as immediate non-oral status or thickening liquids, may be unnecessarily restrictive. Cimoli et al. highlight that recommending NPO status is not a benign nor preventative intervention. Borders and Steele note the importance of recognizing variation within a person's swallow ability, and that altering consistency can impact function both positively and negatively. Research by Cimoli et al. and Okon et al. emphasize that restrictive recommendations could lead to adverse outcomes such as malnutrition, dehydration, decreased quality of life and social isolation. Clinical decisions should be based on a comprehensive assessment of risk factors, rather than responding reflexively to the mere presence of aspiration (Cimoli et al., Ashford).

### Myth 2: Coughing during meals is bad

Coughing during mealtimes often incites fear of a failure in the swallowing mechanism requiring intervention. In reality, coughing provides clues to aid the clinician in improved understanding of swallow function and may drive treatment decisions. A strong cough signals a functional sensorimotor airway defense system, while a weak or absent cough (i.e., hypotussia) indicates the need for further assessment (Cimoli et al.). Dallal-York and Troche provide an overview of the clinician's role in treating hypotussia and present evidence that training cough skill and strength can improve swallow outcomes.

Understanding the protective nature of cough requires reframing how we interpret it during mealtimes and underscores the importance of instrumental evaluation to clearly identify the impact of the cough response. Rather than viewing cough as a red flag, clinicians should consider it within the broader context of an individual's overall status and swallow function (Cimoli et al., Palmer and Padilla). A strong cough response demonstrates intact laryngeal sensation and aids in clearing aspirate from the airway. The goal of treatment is not to eliminate coughing but rather to understand its role in airway protection and see if cough intervention is warranted (Dallal-York and Troche).

### Myth 3: Thickened liquids reduce aspiration risk

Thickened liquids are commonly prescribed to manage dysphagia. Okon et al. note that nursing home staff often thicken liquids based on the belief that they reduce aspiration risk without drawbacks. While there is certainly a place for this recommendation, as thickened liquids can reduce the rate of aspiration, the use of thickened liquids does not always translate to improved clinical outcomes (Okon et al.) and can lead to dehydration, reduced quality of life, decreased medication absorption, and reduced adherence to treatment recommendations. Borders and Steele emphasize the need for an individualized approach to the use of thickened liquids. They note that while thicker consistencies in general are associated with a safer swallow (i.e., improved penetration-aspiration scores), for some individuals, thickened liquids demonstrate worse airway protection. Further, thickening beyond mildly thick does not provide additional safety benefit (Borders and Steele) and if thickened liquids are aspirated, they result in greater damage to the pulmonary system (Palmer and Padilla). Thickened liquids should be viewed as one of many treatment options rather than a sole long-term solution. When employed, thickening recommendations require regular reassessing rather than the common “set it and forget it” approach.

## Conclusion

Managing aspiration risk and its potential complications requires understanding of the multiple factors that can affect outcomes. Prandial aspiration presents only one piece of this complex clinical puzzle. Rather than focusing solely on eliminating aspiration by using restrictive treatment approaches (i.e., non-oral or dietary modifications), we should think broadly and consider the risk of any adverse event to an individual. Aspiration may be tolerated in healthy, medically stable individuals, while a more conservative approach may be needed for people with multiple, non-modifiable risk factors, such as those who are immunocompromised.

The future of aspiration management lies not in seeking universal solutions but rather in developing clinical algorithms that account for both the individual/internal and environmental/external factors. We must respect patient preferences as there is no “one size fits all” approach to managing aspiration given the varied risk profiles across individuals.
